# Determinants of Neonatal Mortality at a Referral Paediatric Hospital in Angola: A Case–Control Study Using Theoretical Frameworks

**DOI:** 10.3390/ijerph21121609

**Published:** 2024-11-30

**Authors:** Israel C. Avelino, Joaquim Van-Dúnem, Luís Varandas

**Affiliations:** 1 Global Health and Tropical Medicine (GHTM), LA-REAL, Instituto de Higiene e Medicina Tropical (IHMT), Universidade NOVA de Lisboa, 1169-056 Lisboa, Portugal; 2 Clínica Multiperfil, Luanda 2177, Angola; 3 Faculty of Medicine, Agostinho Neto University, Luanda 815, Angola; joaquim.vandunen@uan.ao; 4 Nova Medical School, Faculdade de Ciências Médicas, Universidade Nova de Lisboa, 1169-056 Lisboa, Portugal; 5 Departamento de Pediatria, Hospital Dona Estefânia, 1169-045 Lisboa, Portugal

**Keywords:** Angola, neonatal mortality, neonatal care, case–control study

## Abstract

Neonatal mortality rates in developing countries are influenced by a complex array of factors. Despite advancements in healthcare, Angola has one of the highest neonatal mortality rates in sub-Saharan Africa, with significant contributors including premature birth, intrapartum events, tetanus, and sepsis. This study, utilizing key theoretical frameworks such as intersectionality, social determinants of health (SDOH), and ecosocial theory, aimed to identify the primary causes and contributing factors of neonatal mortality among infants admitted to the Neonatology Service at DBPH in Luanda from May 2022 to June 2023. A retrospective matched case–control design was employed, pairing each neonatal death with two surviving neonates based on age and sex. The analysis included 318 newborns, of whom 106 experienced hospital deaths. A stepwise binary logistic regression model was used to examine associations between variables and neonatal mortality. Variables with *p* < 0.25 in bivariate analysis were included in the multivariate model. Significant factors associated with neonatal mortality included the following: a low Apgar score at 1 min (<7) (OR 2.172; 95% CI: 1.436–4.731); maternal age under 20 years (OR 3.746; 95% CI: 2.172–6.459); home delivery (OR 1.769; 95% CI: 1.034–3.027); and duration of illness before admission ≥ 3 days (OR 2.600; 95% CI: 1.317–5.200). Addressing these issues requires urgent interventions, including improving Apgar score management through enhanced training for healthcare professionals, supporting young mothers with intensified maternal education, ensuring deliveries occur in appropriate healthcare settings, and improving universal health coverage and referral systems. These measures could be crucial for enhancing neonatal care and reducing mortality.

## 1. Introduction

Neonatal mortality, defined as death occurring within the first 28 days of life [1], poses a significant challenge to global public health, particularly in developing nations [2,3] with limited resources [4,5,6,7]. Statistics from the World Health Organization (WHO, 2020) indicate that approximately 2.4 million neonatal deaths occur annually, accounting for nearly half of all deaths in children under five years old [4].

Sub-Saharan Africa has recorded the highest neonatal mortality rate, with 27 deaths per 1000 live births in 2019, followed by Central and South Asia with 24 deaths per 1000 live births [8,9]. The likelihood of death within the first month of life is approximately ten times higher in these regions compared to high-income countries [8,10].

Sustainable Development Goal (SDG) 3 has set ambitious targets for 2030, including reducing neonatal mortality to 12 or fewer deaths per 1000 live births [11]. Despite significant reductions in neonatal mortality since 1990, continuous efforts are required to accelerate progress and achieve SDG 3 targets by 2030 [12].

Neonatal mortality rates in developing countries are influenced by a complex interplay of factors. These include access to prenatal care, pregnancy complications, place of delivery, birth weight, neonatal healthcare and overall maternal health indicators. These factors exhibit significant regional and national variability, underscoring disparities in healthcare access and quality.

Angola faces significant challenges in meeting the SDGs, including inadequate healthcare infrastructure, a shortage of qualified health professionals [2,13,14], and limited access to prenatal and postnatal care [14]. Additionally, issues related to extremes of maternal age, poverty, and low levels of caregiver education further complicate the situation [15]. Despite these challenges, the country has made progress in child health, reducing its under-five mortality rate from 105 to 71.5 per 1000 live births between 2012 and 2020 [1,2,8]. However, the neonatal mortality rate remains concerning, at 22 per 1000 live births [2].

The leading causes of neonatal death in Angola include complications from premature birth, intrapartum events, and neonatal sepsis [2,15], underscoring the critical need for quality maternal and neonatal care services to prevent these deaths.

Most studies on neonatal mortality in Angola to date have relied on data from population surveys and general hospital records with a cross-sectional approach [2,6,16,17]. The rationale for this hospital-based case–control study lies in the urgent need to comprehensively understand the determinants of neonatal mortality in Angola.

This study is aligned with the Sustainable Development Goals (SDGs) and utilizes key theoretical frameworks to analyze factors associated with hospital neonatal mortality, with the aim of identifying significant patterns and their public health implications. We employ intersectionality theory to explore how social determinants—such as gender, race, and socioeconomic status—interact to influence neonatal outcomes [18]. This framework helps elucidate the effects of variables like newborn sex, maternal age, and residential location on mortality rates.

In addition, the Social Determinants of Health (SDOH) Framework is applied to investigate how maternal education, access to prenatal care, delivery method, and living conditions impact neonatal mortality. This framework highlights the role of disparities and structural barriers in healthcare access [19,20]. Furthermore, ecosocial theory integrates social, environmental, and biological factors that affect health outcomes, including gestational age, birth weight, and maternal health [21].

By addressing critical gaps in the understanding of neonatal mortality determinants within hospital settings, this study examines the complex interplay between clinical and sociodemographic factors. The findings aim to inform public health policies and improve maternal and child care, particularly in the context of Angola.

## 2. Materials and Methods

### 2.1. Study Design, Type, and Location

A matched case–control study was conducted at the David Bernardino Pediatric Hospital (DBPH) in Luanda, a tertiary-level teaching and research center, and the primary referral facility for pediatric care within Angola’s National Health Service. Operating with 554 inpatient beds across 12 services and maintaining a 97% occupancy rate, DBPH admits neonates primarily from other healthcare facilities due to its lack of a maternity unit. This study investigates neonatal mortality within this specific context, where the clinical profiles of admitted neonates are influenced by prior care at public or private institutions. Data were retrospectively retrieved from the neonatology service records at DBPH’s Statistics Department for the period from May 2022 to June 2023. Factors associated with neonatal deaths were identified based on the relevant literature [14,15,22,23,24], using routinely collected data from the neonates’ medical records.

The Neonatology Service comprises a main ward with 21 beds, a specialized room for the care of premature infants, a dedicated unit for infants with tetanus, and a post-operative care area staffed by a nurse. Each area is equipped to deliver comprehensive care tailored to the specific needs of neonatal patients, providing a secure and supportive environment for their recovery and development. This structured setup is designed to optimize care for neonates with diverse clinical needs and enhance overall patient outcomes.

### 2.2. Population and Sample

The study defined cases as neonates admitted to the neonatology service between May 2022 and June 2023 who died within the first 28 days of life. Controls were neonates admitted to the same service in the same month as the corresponding case’s death and who survived beyond the first 28 days of life. Controls were matched to each case by age (±2 days) and sex (1 case: 2 controls).

### 2.3. Inclusion and Exclusion Criteria

All neonatal cases recorded in the service’s registry book, matched by age and sex with two control neonates each, were included and analyzed. Neonates with incomplete records or insufficient information for the study’s objectives were excluded, as were those with unknown discharge outcomes or unknown dates of birth.

### 2.4. Sample Size Estimation

Sample size was determined using G*Power software (Version 3.1.9.7, Heinrich-Heine-Universität Düsseldorf, Germany) [25]. Based on hospital statistics from the previous year, the following parameters were used: a 95% confidence interval, 90% power, a proportion of non-institutional births among cases (P1) of 63.4%, and among controls (P0) of 44.1%. The minimum detectable odds ratio was set at 2.2 with a case-to-control ratio of 1:2, and a phi coefficient of 0.2 was assumed for the correlation coefficient (r) between cases and controls [26]. The estimated minimum sample size was initially 276 (92 cases and 184 controls), which was increased by 15% to account for contingencies, resulting in a final sample of 318 participants (106 cases and 212 controls).

### 2.5. Sample Selection

A simple random sampling approach was employed to ensure the representativeness of the case and control groups and to enable valid comparative analyses. The medical records department provided files of cases and controls based on the neonatal hospital admission list during the study period, which comprised 3653 admissions, including 527 deaths. Each neonatal death case was assigned a unique number, and cases were selected using an electronic random number generator until the desired sample size was achieved, ensuring that each newborn had an equal chance of being included in the case group. Similarly, two controls were randomly selected for each case from patients discharged to home, matched by age (±2 days of the corresponding case) and sex. If a generated control did not meet the matching criteria, another number was generated until a suitable match was found. During this process, 16 records (5 cases and 11 controls) were excluded.

### 2.6. Data Collection and Questionnaires

A structured data collection instrument was used to assess factors associated with neonatal death by reviewing medical records. The instrument included only variables routinely collected during newborn admissions. The following components were considered:

- Dependent variable: neonatal mortality.
- Independent variables: demographic and biological data, including gestational age, newborn sex, birth weight, Apgar score at one minute, Apgar score at five minutes, and neonate’s age.
- Socioeconomic factors: maternal age, maternal parity, place of residence.
- Prenatal care and delivery: type of delivery, place of delivery, number of prenatal visits, duration of illness before admission.
- Exposures and health conditions: maternal HIV status, breastfeeding, vaccinations, umbilical cord treatment.

The Data Collection Instrument and the Operational Definition of Variables are presented as Supplementary Material (File S1).

### 2.7. Data Quality Assurance Techniques, Data Processing, and Analysis

Two data collectors participated in the process. They underwent a two-day training session conducted by the principal investigator on the checklist and data extraction procedures. Prior to actual data collection, a pilot testing phase was conducted on 5% of the checklists to ensure adequacy. Following data collection, a rigorous validation process was implemented where 10% of the data entries were cross-checked against medical records to ensure data consistency. Subsequently, the coded data underwent thorough cleaning to address missing values and variables. Data analysis was performed using IBM SPSS Statistics for Windows, version 26.0 (IBM Corp.^®^, Armonk, NY, USA) under the supervision of the principal investigator.

### 2.8. Statistical Analysis

Statistical analysis was conducted in two stages. The initial descriptive analysis summarized quantitative variables using the median and interquartile range (IQR), given their non-normal distribution (*p* = 0.001). Categorical variables were described using absolute and relative frequencies, with cutoff points derived from the literature. The proportionate mortality rate (PMR) was calculated by dividing the number of cause-specific deaths by the total number of deaths and then multiplying by 100. The effect size of explanatory variables was estimated using the crude Odds Ratio (OR) with 95% Confidence Intervals (CI). In the second stage, a stepwise binary logistic regression model was used to examine the association between each independent variable and the dependent variable (neonatal mortality). Variables with a significance level of *p* < 0.25 in bivariate analysis were considered for inclusion in the multivariate model. Interaction terms were included in the logistic regression model to evaluate the significance of combined effects of maternal age with newborn sex and place of residence on neonatal mortality. Model adequacy was assessed using the Hosmer–Lemeshow goodness-of-fit test, and collinearity among independent variables was evaluated with variance inflation factors (VIF), revealing no significant multicollinearity. Adjusted Odds Ratios (AOR) with 95% CI were computed to quantify the strength of associations, with statistical significance set at *p* < 0.05 for neonatal mortality. Finally, the results were presented using tables and narrative text.

All maternal and neonatal factors were extracted from the Neonatal Intensive Care Unit (NICU) registry and hospital statistics department records. The International Classification of Diseases (ICD-10) was utilized to classify the underlying causes of neonatal deaths.

## 3. Results

### Sample Characterization

**Figure 1:**
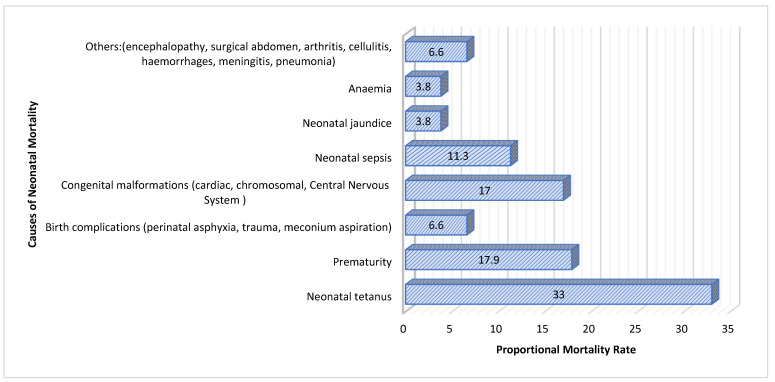
Proportional mortality rate by cause.

A total of 318 medical records (106 cases and 212 controls) were retrospectively reviewed from May 2022 to June 2023, with a slight predominance of females (57.9%). Maternal age had a median (IQR) of 22 (10) years, while the newborns included in the study had a median (IQR) age of 9 (9) days, ranging from 1 to 28 days. The median (IQR) length of hospital stay was 4 (4) days, with durations ranging from 1 to 24 days. The median (IQR) birth weight of the neonates was 2880 (955) grams, with weights ranging from 800 g to 5000 g. Among the cases, 56 (52.8%) were born in a hospital setting, while 50 (47.2%) were born at home or in an ambulance. In contrast, among the controls, 137 (64.6%) were born in a hospital. The characterization of the sample is detailed in Table 1, Table 2, Table 3, Table 4 and Table 5, as well as in the accompanying figure (Figure 1). 

The logistic regression analysis (Table 6) identified key factors significantly associated with neonatal mortality: Apgar score < 7 (OR = 2.607), maternal age < 20 years (OR = 3.746), home delivery (OR = 1.769), and duration of illness before admission ≥ 3 days (OR =2.560). All these factors were significantly associated with an increased risk of neonatal mortality during the study period.

Further interaction analysis showed that the combination of maternal age < 20 years and rural residence significantly increased the risk of neonatal mortality, with younger mothers in rural areas having a 2.333-fold higher risk compared to older mothers or those residing in urban areas (B = 0.845, *p* = 0.009).

## 4. Discussion

This case–control study conducted at DBPH in Angola identified neonatal tetanus as the primary cause of neonatal mortality, with a Proportionate Mortality Ratio (PMR) of 33.0. Neonatal tetanus, preventable through proper umbilical cord care and vaccination, remains a critical issue due to insufficient vaccination coverage and inadequate hygiene practices [2,17,27]. Addressing these factors by improving vaccination rates and hygiene practices is essential to reducing neonatal mortality from tetanus.

Premature infants are at increased risk of severe complications and mortality, highlighting the need for enhanced neonatal intensive care and postnatal support [28,29]. The recent literature emphasizes that interventions to improve intensive care capabilities and promote full-term deliveries are crucial for better outcomes [29,30]. Additionally, birth complications, such as perinatal asphyxia and trauma (PMR of 6.6), and congenital malformations (PMR of 17.0), significantly contributed to neonatal mortality. This underscores the importance of implementing safe delivery practices and early diagnosis and management of congenital conditions [31,32,33].

Neonatal sepsis and jaundice were other major causes of mortality. Sepsis, associated with severe infections, necessitates improvements in infection control and treatment protocols. Although less prevalent, neonatal jaundice requires timely monitoring and treatment to prevent severe outcomes [34]. Implementing effective protocols and enhancing neonatal care practices are vital for reducing mortality rates and improving neonatal outcomes.

Infants with an Apgar score of <7 at 1 min had a two-fold higher risk of mortality compared to those with a score of ≥7. This finding aligns with the existing literature, which correlates low Apgar scores with adverse outcomes such as perinatal asphyxia and respiratory complications, which are associated with higher mortality rates [35,36,37].

Low birth weight (LBW) is a well-established predictor of neonatal mortality and morbidity, particularly in resource-limited settings where care may be suboptimal [38,39,40]. This study supports the existing literature, indicating that the risk of neonatal death is 1.6 times higher in infants with LBW. Conversely, the association with macrosomia was not statistically significant; however, it remains important to monitor due to potential implications for neonatal health [40]. These findings highlight the critical need for public health policies aimed at reducing LBW and improving neonatal care, which are essential for mitigating mortality and enhancing health outcomes in vulnerable populations.

Maternal age also emerged as a significant determinant of neonatal mortality. Mothers under 20 years of age faced a risk of neonatal mortality 3.5 times higher compared to those aged 20 to 35 years. This finding is consistent with the literature linking adolescent pregnancies to higher rates of neonatal complications due to factors such as biological immaturity and limited access to prenatal care [3,41,42,43,44]. In contrast, mothers aged 35 years or older exhibited a reduced risk compared to the reference group. While advanced maternal age is often associated with specific risks, these risks did not significantly exceed those observed in the reference group, consistent with recent studies highlighting the complexities of pregnancies at older ages [43,45,46].

Parity was also identified as a significant factor. Mothers with four or more children had a neonatal mortality risk 2.3 times higher compared to those with three or fewer children. This result is consistent with the literature associating high parity with adverse neonatal outcomes. While some studies suggest that multiparity may be associated with lower risks for certain adverse birth outcomes [47], higher birth orders are frequently linked with complications such as prolonged gestation, increased likelihood of preterm birth, and reduced access to adequate prenatal care, all of which contribute to increased neonatal mortality [48,49]. Additionally, high parity may reflect lower resource availability and social support, which are crucial for healthy pregnancies and positive neonatal outcomes [43,50].

Residential location, whether rural or urban, also demonstrated a significant association with neonatal mortality. The risk was 2.5 times higher for residents of rural areas compared to their urban counterparts. This increased risk can be attributed to several factors, including reduced access to quality healthcare, lower availability of emergency services, and limited resources in rural areas [51,52]. Disparities in healthcare access between urban and rural areas significantly impact neonatal outcomes, highlighting the need for health policies that address these inequalities [53,54]. Additionally, the interaction between young maternal age and place of residence reveals an even greater risk of neonatal mortality. The trend concerning maternal age and newborn sex further emphasizes the vulnerability of male infants born to younger mothers, highlighting potential difficulties in providing adequate care during the neonatal period.

Prenatal care and delivery conditions were also assessed. The type of delivery did not show a significant association with neonatal mortality, consistent with mixed results in the literature regarding delivery methods. While some studies suggest cesarean sections may be linked to increased neonatal complications, others indicate that appropriately indicated cesarean deliveries can mitigate these risks [55,56,57,58].

Conversely, the place of delivery reveals a statistically significant association with neonatal mortality (*p* = 0.04), with home deliveries associated with a 1.6-fold higher risk compared to hospital deliveries. This underscores the importance of hospital settings, which provide essential medical resources and skilled personnel [59,60]. Additionally, a longer illness duration before hospitalization (≥3 days) was significantly associated with an increased risk of neonatal death (OR: 2.6), emphasizing the critical importance of early recognition of severe illness and timely clinical intervention. These findings are consistent with the existing literature, which underscores the importance of enhancing parental education and improving access to healthcare services as key factors in reducing neonatal mortality [2,53,54]. Delays in accessing medical care, often due to reliance on home remedies [61], or limited availability of timely healthcare, likely contributed to poorer outcomes.

The association between maternal HIV status and neonatal mortality yielded an Odds Ratio (OR) of 2.5 (95% CI: 0.7–8.3), suggesting a potential increased risk for infants born to HIV-positive mothers. Although this finding did not reach statistical significance, it is consistent with the existing literature linking maternal HIV infection to adverse neonatal outcomes, such as vertical transmission and compromised maternal health [62,63]. This underscores the critical need for enhanced maternal healthcare interventions to mitigate these risks and improve neonatal survival rates.

The likelihood of neonatal death was 4.2 times higher among non-breastfed infants. Current research highlights the substantial protective effect of breastfeeding against neonatal mortality, due to its provision of essential nutrients and antibodies critical for newborn health [64,65].

Vaccination status also revealed a significant association with neonatal mortality, with unvaccinated neonates exhibiting an OR of 1.8 (95% CI: 1.1–2.9), indicating that they are 1.8 times more likely to die compared to their vaccinated counterparts. The absence of vaccination increases the vulnerability of these infants to severe, preventable infectious diseases, including tuberculosis meningitis, miliary tuberculosis, and poliomyelitis, which are major contributors to neonatal mortality [66,67,68,69]. In addition, inadequate umbilical cord care was linked to a two-fold increased risk of neonatal death. Proper care of the umbilical cord is vital for preventing infections like sepsis and other complications. Effective management requires collaboration between healthcare providers and mothers to ensure optimal neonatal outcomes [61,70].

The findings of this study are consistent with theoretical frameworks such as the ecosocial theory and intersectionality, which emphasize the complex interplay between individual, social, and structural factors in shaping health outcomes. For instance, maternal age and residential location are not merely biological or demographic characteristics, but are embedded within broader social structures that influence access to healthcare, education, and economic resources. The higher neonatal mortality risk associated with younger mothers, especially those living in rural areas, highlights the intersectionality of youth, gender, and geographical location. In rural areas, these young mothers often face barriers such as limited access to prenatal care, health literacy, and timely medical intervention, all of which are critical in ensuring healthy neonatal outcomes.

Additionally, factors such as low birth weight (LBW) and premature birth resonate with the ecosocial framework, which posits that early life conditions, compounded by socioeconomic disadvantage, significantly influence health trajectories. The study’s findings of a higher neonatal mortality risk in LBW infants emphasize the need for enhanced maternal care, nutrition, and infection prevention, particularly in resource-limited settings like Angola. Similarly, the link between inadequate vaccination and neonatal mortality underscores the role of SDOH, such as access to healthcare—especially in rural areas—as a critical determinant of both maternal and neonatal outcomes.

### Limitations

This study is limited by its retrospective design and reliance on available data, which limits our ability to control for key confounding factors influencing neonatal mortality, such as birth conditions, paternal age, and family structure. Additionally, we did not account for assessing the severity on admission using objective clinical measures, nor did we evaluate the quality of neonatal intensive care and follow-up services, thus restricting our ability to assess their impact on mortality rates.

Although this study identified several key factors associated with neonatal mortality, certain unaccounted confounders, such as paternal age and family structure, may have influenced the results. For example, paternal age could indirectly affect neonatal health by influencing maternal health, socioeconomic status, and access to healthcare. Similarly, aspects of family structure—such as single motherhood or the absence of social support—may impact maternal well-being and, consequently, neonatal outcomes. Although these variables were not considered in this analysis, future research should include them to provide a more comprehensive understanding of the determinants of neonatal mortality.

The setting of our study at DBPH, a tertiary referral center that primarily manages severe neonatal cases, may lead to an overestimation of mortality rates, as it reflects case severity rather than the overall quality of care. The hospital’s lack of a maternity unit means that neonates are admitted only after being referred from other facilities, excluding those born at home or in non-specialized hospitals. Consequently, our sample may not be representative of the general neonatal population in Angola, as it disproportionately includes high-risk cases. Furthermore, by focusing solely on referred cases, we excluded neonatal deaths occurring outside the referral network, such as those in rural areas or smaller hospitals. This introduces selection bias and limits the generalizability of our findings, as they may not fully capture neonatal outcomes across the broader range of healthcare settings in Angola.

Finally, the high occupancy rate of 97% at DBPH raises concerns about potential referrals being declined, further affecting the sample’s representativeness. Despite these limitations, our findings highlight the urgent need for targeted interventions and better contextualization of DBPH data to inform health policies and improve neonatal outcomes.

## 5. Conclusions

This study, conducted at David Bernardino Paediatric Hospital (DBPH), has identified critical determinants of neonatal mortality based on a thorough analysis rooted in robust theoretical frameworks. The key predictors of neonatal mortality identified were the following: Apgar score at one minute (<7), maternal age under 20 years, home delivery, and illness duration before hospital admission ≥ 3 days. These factors underscore the urgent need for targeted interventions, including improved management of low Apgar scores, enhanced support for young mothers, ensuring deliveries occur in appropriate healthcare settings, and strengthening referral and counter-referral systems. Furthermore, the study underscores the importance of early medical intervention and the implementation of more effective screening processes at the point of admission.

## 6. Recommendations

To address these challenges, we recommend several strategies. (1) Capacity building for healthcare professionals: develop and implement training programs focused on neonatal resuscitation and postnatal care to improve outcomes related to low Apgar scores. (2) Intensified maternal education: initiate educational campaigns aimed at young pregnant women to highlight the importance of antenatal care and the risks associated with early pregnancies. (3) Improvement in access to healthcare services: invest in infrastructure to ensure all deliveries occur in hospital settings, including the establishment of community health centers and facilitation of transportation for pregnant women. (4) Optimization of the healthcare system: integrate primary healthcare services, alternative treatment institutions, and hospitals to ensure efficient referrals and expand primary care for early detection of neonatal conditions. (5) Evidence-based policy formulation: utilize this study’s findings to inform public policies that prioritize maternal and neonatal health, ensuring that interventions are grounded in data and contextually relevant. Implementing these recommendations can significantly reduce neonatal mortality and drive substantial improvements in maternal and child health in Angola. Further research should also focus on identifying objective measures of illness severity that can be assessed upon admission, which could enhance risk stratification and guide clinical decision-making.

## Figures and Tables

**Table 1:** Frequency distribution of diagnoses for cases (cause of death) and controls (hospital discharge).

ICD-10 Diagnosis	Diagnosis	Case n (%)	Control n (%)	Total n (%)
A33	Neonatal tetanus	35 (33.0)	18 (8.5)	53 (16.7)
P07	Prematurity	19 (17.9)	22 (10.4)	41 (12.9)
P21–P24	Birth complications (perinatal asphyxia, trauma, meconium aspiration)	7 (6.6)	8 (3.8%)	15 (4.7)
Q00–Q99	Congenital malformations (cardiac, chromosomal, central nervous system)	18 (17.0)	70 (33.0)	88 (27.7)
P36	Neonatal sepsis	12 (11.3)	33 (15.6)	45 (14.1)
P59	Neonatal jaundice	4 (3.8)	30 (14.2)	34 (10.7)
P61	Anaemia	4 (3.8)	7 (3.3)	11 (3.5)
Others	(encephalopathy, surgical abdomen, arthritis, cellulitis, haemorrhages, meningitis, pneumonia)	7 (6.6)	24 (11.3)	31 (9.7)
Total		106 (100)	212 (100)	318 (100)

**Table 2:** Association between demographic and biological factors and neonatal mortality.

Variable	Cases (Number/%)	Controls (Number/%)	OR (95% CI)
Gender			
Male	45 (42.5)	90 (42.5)	1.0 (0.6–1.6)
Female	61 (57.5)	122 (57.5)	1
Neonate’s age			
≤7 days	49 (46.2)	79 (37.3)	1.5 (0.9–2.3)
8–28 days	57 (53.8)	133 (62.7)	1
Gestational age			
Preterm	28 (26.4)	37 (17.5)	1.7 (1.0–30)
Term	76 (71.7)	170 (80.2)	1
Post-Term	2 (1.9)	5 (2.3)	0.9 (0.2–4.7)
Birth weight			
Low Birth Weight	42 (39.6)	59 (27.8)	1.6 (1.0–2.7)
Normal Birth Weight	61 (57.6)	140 (66.1)	1
Macrosomia	3 (2.8)	13 (6.1)	0.5 (0.1–1.9)
1st minute Apgar			
<7	35 (33.0)	42 (19.8)	2 (1.2–3.4)
≥7	71 (67.0)	170 (80.2)	1
5th minute Apgar			
<7	11 (10.4)	15 (7.7)	1.5 (0.6–3.4)
≥7	95 (89.6)	197 (92.3)	1

**Table 3:** Association between socioeconomic factors and neonatal mortality.

Variable	Cases (Number/%)	Controls (Number/%)	OR (95% CI)
Maternal age			
<20 years	60 (56.6)	56 (26.4)	3.5 (2.1–5.8)
20–35 years	41 (38.7)	133 (62.7)	1
≥35 years	5 (4.7)	23 (10.8)	0.7 (0.3–2)
Maternal parity			
≤3	97 (91.5)	175 (82.5)	1
≥4	9 (8.5)	37 (17.5)	2.3 (1.1–4.9)
Place of residence			
Urban	100 (94.3)	184 (86.8)	1
Rural	6 (5.7)	28 (13.2)	2.5 (1.1–6.3)

**Table 4:** Association of Prenatal Care and Delivery Factors with Neonatal Mortality.

Variable	Cases (Number/%)	Controls (Number/%)	OR (95% CI)
Type of delivery			
- Cesarean	8 (7.5)	14 (6.6)	1.2 (0.5–2.8)
- Vaginal	98 (92.5)	198 (93.4)	1
Place of delivery			
- Home	50 (47.2)	75 (35.4)	1.6 (1–2.6)
- Hospital	56 (52.8)	137 (64.6)	1
Number of prenatal visits			
<4	70 (66.0)	141 (66.5)	1.0 (0.6–1.6)
≥4	36 (34.0)	71 (33.5)	1
Illness Duration before Admission			
Less than 1 day	10 (9.4%)	50 (23.6%)	1
1 to 2 days	12 (11.3%)	30 (14.2%)	1.6 (0.6–4.1)
3 days or more	84 (79.2%)	132 (62.3%)	2.6 (1.3–5.2)

**Table 5:** Association of exposures and health conditions with neonatal mortality.

Variable	Cases (Number/%)	Controls (Number/%)	OR (95% CI)
Mother HIV status			
- Yes	6 (5.7)	5 (2.4)	2.5 (0.7–8.3)
- No	100 (94.3)	207 (97.6)	1
Breastfeeding			
- No	6 (5.7)	3 (1.4)	4.2 (1.0–17.1)
- Yes	100 (94.3)	209 (98.6)	1
Vaccinations			
- No	55 (51.9)	79 (37.3)	1.8 (1.1–2.9)
- Yes	51 (48.1)	133 (62.7)	1
Umbilical cord treatment			
- Not correct	57 (53.8)	78 (36.8)	2 (1.2–3.2)
- Correct	49 (46.2)	134 (63.2)	1

**Table 6:** Results of logistic regression analysis using the forward stepwise method.

		B	S.E.	Wald	df	Sig.	Exp(B)	95% C.I. for EXP(B)	
								Lower	Upper
Step1 ^a^	Maternal age			26.814	2	0.001			
	<20 years	1.246	0.258	23.361	1	0.001	3.476	2.097	5.760
	≥35 years	−0.349	0.525	0.443	1	0.506	0.705	0.252	1.972
	Constant	−1.177	0.179	43.398	1	0.001	0.308		
Step 2 ^b^	Apgar at 1 min (<7)	0.841	0.287	8.599	1	0.003	2.319	1.322	4.068
	Maternal age			28.457	2	0.001			
	<20 year	1.329	0.265	25.128	1	0.001	3.776	2.246	6.349
	≥35 years	−0.311	0.531	0.342	1	0.558	0.733	0.259	2.075
	Constant	−1.434	0.207	48.147	1	0.001	0.238		
Step 3 ^c^	Apgar at 1 min (<7)	0.860	0.290	8.787	1	0.003	2.364	1.338	4.176
	Maternal age			26.853	2	0.001			
	<20 years	1.324	0.269	24.289	1	0.001	3.759	2.220	6.366
	≥35 years	−0.193	0.535	0.131	1	0.718	0.824	0.289	2.350
	Illness Duration Before Admission (≥3 days)	0.834	0.279	8.910	1	0.003	2.31	1.330	3.970
	Constant	−1.558	0.217	51.753	1	0.001	0.211		
Step 4 ^d^	Apgar at 1 min (<7)	0.902	0.293	9.469	1	0.002	2.464	1.387	4.375
	Maternal age			23.812	2	0.001			
	<20 years	1.267	0.271	21.796	1	0.001	3.550	2.086	6.042
	≥35 years	−0.142	0.539	0.070	1	0.792	0.867	0.301	2.497
	Illness Duration Before Admission (≥3 days)	0.950	0.268	12.560	1	0.003	2.59	1.530	4.380
	Umbilical cord treatment (Not correct)	0.578	0.260	4.928	1	0.026	1.782	1.070	2.969
	Constant	−1.811	0.252	51.477	1	0.001	0.163		
Step 5 ^e^	Apgar at 1 min (<7)	0.852	0.297	8.252	1	0.004	2.344	1.311	4.191
	Maternal age			25.040	2	0.001			
	<20 years	1.326	0.275	23.175	1	0.001	3.764	2.194	6.458
	≥35 years	−0.087	0.539	0.026	1	0.872	0.917	0.319	2.637
	Place of residence (Rural)	−0.959	0.488	3.857	1	0.050	0.383	0.147	0.998
	Illness Duration Before Admission (≥3 days)	0.930	0.257	13.120	1	0.002	2.530	1.530	4.200
	Umbilical cord treatment (Not correct)	0.567	0.263	4.663	1	0.031	1.763	1.054	2.949
	Constant	−1.723	0.255	45.689	1	0.001	0.179		
Step 6 ^f^	Apgar at 1 min (<7)	0.958	0.304	9.923	1	0.002	2.607	1.436	4.731
	Maternal age			24.314	2	0.001			
	<20 years	1.321	0.278	22.566	1	0.001	3.746	2.172	6.459
	≥35 years	−0.072	0.540	0.018	1	0.894	0.930	0.323	2.679
	Place of residence (Rural)	−1.024	0.492	4.339	1	0.037	0.359	0.137	0.941
	Place of delivery (Home)	0.570	0.274	4.327	1	0.038	1.769	1.034	3.027
	Illness Duration Before Admission (≥3 days)	0.940	0.257	13.38	1	0.002	2.56	1.550	4.200
	Umbilical cord treatment (Not correct)	0.498	0.266	3.498	1	0.061	1.646	0.976	2.775
	Constant	−1.944	0.282	47.490	1	0.001	0.143		
interactions	Maternal Age × Newborn Sex (Male)	0.400	0.250	2.500	1	0.113	1.491	0.910	2.440
	Maternal Age × Place of Residence (Rural)	0.845	0.320	6.784	1	0.009	2.333	1.216	4.474

^a^ Variables added in step 1: maternal age; ^b^ variables added in step 2: APGAR at 1 min; ^c^ variables added in step 3: duration of illness before admission; ^d^ variables added in step 4: umbilical cord treatment; ^e^ variables added in step 5: place of residence; ^f^ variables added in step 6: place of delivery.

## Data Availability

The data used in this study belong to DBPH and are managed by IHMT. They may be available upon request to researchers who meet the criteria for accessing confidential data. Data requests should be directed to the appropriate contact at IHMT, and requestors must provide details regarding the purpose of the request and comply with the requirements set forth by the Scientific Council of IHMT. Data should only be used for the purpose defined in the request.

## Supplementary Materials

The following supporting information can be downloaded at: https://www.mdpi.com/article/10.3390/ijerph21121609/s1, File S1: The Data Collection Instrument and the Operational definition of variables.
