# Supplementary material for: Determinants of Neonatal Mortality at a Referral Paediatric Hospital in Angola: A Case–Control Study Using Theoretical Frameworks
Source: Int J Environ Res Public Health. 2024 Nov 30;21(12):1609. doi: 10.3390/ijerph21121609 (PMC11675793; doi:10.3390/ijerph21121609)
Supplement: Supplementary file 1 [file ijerph-21-01609-s001.zip › ijerph-3271610-supplementary.pdf]

Determinants of Neonatal Mortality at a Referral Paediatric Hospital in Angola: A Case-Control Study Using Theoretical Frameworks

Israel C. Avelino, Joaquim Van–Dúnem, Luís Varandas

Data Collection Form

1. Sex

- Question: *What is the sex of the newborn?*
  - ☐ Male
  - ☐ Female

2. Status at Discharge

- Question: *What is the status of the newborn at discharge?*
  - ☐ Case
  - ☐ Control

3. Gestational Age

- Question: *What is the gestational age of the newborn, in completed weeks?*
  - ☐ Less than 37 weeks (Preterm)
  - ☐ Between 37 and 42 weeks (Term)
  - ☐ More than 42 weeks (Post-term)

4. Birth Weight

- Question: *What was the birth weight of the newborn, in grams?*
  - ☐ Less than 2500 grams (Low birth weight)
  - ☐ Between 2500 and 4000 grams (Normal birth weight)
  - ☐ More than 4000 grams (High birth weight)

5. Apgar Score

- Question: *What was the Apgar score of the newborn at one minute and five minutes after birth?*
  - Apgar at 1 minute: [ ] (Field to fill in with number)
  - Apgar at 5 minutes: [ ] (Field to fill in with number)

6. Crying at Birth

- Question: *Did the newborn cry at birth?*
  - ☐ Yes
  - ☐ No

7. Neonatal Age

- Question: *What is the neonatal age of the newborn, in completed days?*
    - ☐ (Field to fill in with number)

8. Time, in days, between the onset of illness and hospital admission

[ ] Less than 1 day (less than 24 hours)
[ ] 1 to 2 days
[ ] 3 days or more

09. Total Length of Hospital Stay

- Question: *What is the total length of hospital stay, in days?*
    - ☐ (Field to fill in with number)

10. Maternal Age

- Question: *What is the mother's age at the time of delivery, in years?*
    - ☐ Under 20 years
    - ☐ Between 20 and 35 years
    - ☐ 35 years or older

11. Maternal Parity

- Question: *How many previous pregnancies has the mother had, including the current one?*
    - ☐ Fewer than 3 pregnancies
    - ☐ Three or more pregnancies

12. Place of Residence

- Question: *What is the mother's place of residence?*
    - ☐ Rural
    - ☐ Urban

13. Type of Delivery

- Question: *What was the method of delivery used for the baby?*

- ☐ Normal (Vaginal)
- ☐ Caesarean

14. Place of Delivery

- Question: *Where did the delivery of the baby take place?*
  - ☐ Hospital
  - ☐ At home

15. Number of Antenatal Consultations

- Question: *How many antenatal consultations were conducted during the pregnancy?*
  - ☐ Fewer than 4 consultations (Inadequate)
  - ☐ 4 or more consultations (Adequate)

16. Maternal HIV Status

- Question: *What is the mother's HIV status during pregnancy?*
  - ☐ HIV positive
  - ☐ HIV negative

17. Breastfeeding

- Question: *Is the newborn being exclusively breastfed since birth?*
  - ☐ Yes
  - ☐ No

18. Vaccinations

- Question: *Did the mother receive all the recommended vaccinations during pregnancy?*
  - ☐ Received
  - ☐ Not received
- Question: *Did the newborn receive all the recommended vaccinations at birth?*
  - ☐ Received
  - ☐ Not received

19. Umbilical Cord Care

- Question: *How was the umbilical cord care managed after birth?*
  - ☐ Adequate (Use of antiseptics, such as 70% ethanol)

  - ☐ Inadequate (Use of substances such as salt, palm oil, clay, leaves, and other non-medical materials)

20. Diagnosis

- Question: *What is the diagnosis of the newborn?*
  - ☐ (Field to fill in with diagnosis)

Israel C. Avelino, Joaquim Van–Dúnem, Luís Varandas

Operational definitions of variables

Variable	Definition	Categorization
Gestational Age	The duration of pregnancy, expressed in completed weeks, calculated from the first day of the mother's last menstrual period or confirmed by ultrasound.	Preterm: Less than 37 weeks gestation. Term: Between 37 and 42 weeks gestation. Post-term: More than 42 weeks gestation.
Birth Weight	The weight of the newborn measured shortly after birth using a calibrated scale, recorded in grams.	Low Birth Weight: Less than 2500 grams; Appropriate Birth Weight: Between 2500 and 4000 grams; High Birth Weight: Above 4000 grams
Apgar Scores	Assessment of the newborn's vital signs by a healthcare professional at the first and fifth minutes after birth.	Apgar ≥7: Adequate adaptation; Apgar <7: Requires additional attention
Neonate Age	The age of the newborn calculated in complete days from the exact date and time of birth.	≤ 7 days (Early neonatal); 8 – 28 days (Late neonatal).
Maternal Age	The age of the mother at the time of delivery.	<20 years; 20–35 years; ≥ 35 years
Maternal Parity	Number of previous pregnancies lasting more than 20 weeks, including the current delivery [50].	Less than 3 previous pregnancies; Three or more previous pregnancies
Place of Residence	The geographical location where the mother resides, as defined by official administrative boundaries.	Rural or Urban
Type of Delivery	Method used for the baby's delivery.	Normal (Vaginal) or Cesarean
Place of Delivery	Setting where the baby's birth occurs.	Hospital or Home
Number of Antenatal Visits	The total number of prenatal care visits attended by the mother during pregnancy, with the WHO recommending a minimum of four visits for adequate care.	< 4 visits (inadequate); ≥ 4 visits (adequate)

Maternal HIV Status	Presence or absence of HIV infection in the mother during pregnancy. Determined by specific laboratory tests during pregnancy or before delivery.	HIV positive: Presence of HIV infection in the mother during pregnancy; HIV negative: Absence of HIV infection in the mother during pregnancy
Breastfeeding	Practice of exclusively feeding the newborn with breast milk in the first months of life.	(Yes/No)
Vaccinations	The record of administration of vaccines recommended by national and international health guidelines during pregnancy and for the newborn.	Received: Record of administration of recommended vaccines for the newborn; Not received: Absence of record of administration of recommended vaccines
Umbilical Cord Care	The practice and technique used for the care of the umbilical cord stump after birth, including cleaning and monitoring to prevent infection.	Appropriate (The use of antiseptics, such as 70% ethyl alcohol) or Inappropriate (The application of substances like salt, palm oil, clay, leaves, and other non medical materials).
Illness Duration after Admission	Refers to the length of time, measured in hours, that a neonate remains hospitalized due to critical health conditions present at the time of admission.	**≤ 48 hours:** Neonates who are admitted and remain in the hospital for 48 hours or less before mortality occurs.<br><br>**> 48 hours:** Neonates who remain hospitalized for more than 48 hours, indicating a less severe initial condition or the potential for recovery.
